# Lifestyle intervention for metabolic dysfunction-associated fatty liver disease: a 24-h integrated behavior perspective

**DOI:** 10.1007/s12072-024-10663-9

**Published:** 2024-05-08

**Authors:** Shelley E. Keating, Yogesh Chawla, Arka De, Elena S. George

**Affiliations:** 1 School of Human Movement and Nutrition Sciences, The University of Queensland, Brisbane, QLD Australia; 2 Kalinga Institute of Medical Sciences (KIMS), Bhubaneshwar, India; 3 Postgraduate Institute of Medical Education and Research, Chandigarh, India; 4 Institute for Physical Activity and Nutrition, School of Exercise and Nutrition Sciences, Deakin University, Geelong, Australia

**Keywords:** Exercise, Diet, Nutrition, MAFLD, Mediterranean diet, Physical activity

## Abstract

**Introduction:**

The prevalence, health and socioeconomic burden of metabolic dysfunction-associated fatty liver disease (MAFLD) is growing, increasing the need for novel evidence-based lifestyle approaches. Lifestyle is the cornerstone for MAFLD management and co-existing cardiometabolic dysfunction. The aim of this review was to evaluate the evidence for lifestyle management of MAFLD, with a specific lens on 24-hour integrated behaviour and provide practical recommendations for implementation of the evidence.

**Results:**

Weight loss ≥ 7–10% is central to lifestyle management; however, liver and cardiometabolic benefits are attainable with improved diet quality and exercise even without weight loss. Lifestyle intervention for MAFLD should consider an integrated ‘24-h’ approach that is cognisant of diet, physical activity/exercise, sedentary behavior, smoking, alcohol intake and sleep. Dietary management emphasises energy deficit and improved diet quality, especially the Mediterranean diet, although sociocultural adaptations to meet preferences should be considered. Increasing physical activity and reducing sedentary behavior can prevent MAFLD, with strongest evidence in MAFLD supporting regular structured moderate–vigorous aerobic exercise for 150–240 min/week. Resistance training in addition to aerobic exercise should be considered and prioritised for those who are losing body mass via diet and/or pharmacological approaches and those with sarcopenia, to minimise bone and lean mass loss. Limited evidence suggests that sleep is important for MAFLD prevention. Emerging novel approaches to diet and exercise may address some of the key barriers to behaviour change (e.g. lack of time, access to resources and social support).

**Future Directions:**

Large-scale multidisciplinary trials in people with MAFLD with long-term follow-up, that can be scaled up into mainstream healthcare, are required. Future management guidelines should consider the heterogeneity of MAFLD and specialised models of care that coordinate the health workforce to manage the increased and growing MAFLD population.

## Introduction

Metabolic dysfunction-associated fatty liver disease (MAFLD) is the most common liver disease worldwide with most recent estimates as high as 39% in adults and 20% in children [1, 2]. Characterised by hepatic steatosis in the setting of overweight/obesity, type 2 diabetes and/or metabolic dysregulation, MAFLD may co-exist with a broad range of comorbid liver and cardiometabolic conditions [3]. The clinical and public health implications of the updated definition (from non-alcoholic fatty liver disease [NAFLD]) to MAFLD have been described [1, 4, 5]. Notably, those with MAFLD have a heightened risk of cardiovascular disease and overall mortality and are more likely to experience poorer disease prognosis [1, 4, 5]. People with MAFLD are likely to have more complex needs and require integrated multidisciplinary care to adopt and maintain healthy lifestyle behaviors. Moreover, as people with MAFLD do not constitute a homogenous group, rather several subtypes of the disease can be distinguished, there is an increased need for individualised specialist care. The higher prevalence of MAFLD exacerbates issues such as insufficient healthcare workforce. Prevention, community-based screening, early diagnosis and multidisciplinary management of MAFLD are pivotal for a public health response. The aim of this review is to provide clinical guidance for the lifestyle management of MAFLD in adults based on established and emerging evidence. It also aims to explore contemporary opportunities for lifestyle management with a specific lens on a ‘24-h’ integrated approach including diet, exercise, sedentary behavior, substance use and sleep. Whilst there is some overlap in definitions, most of the literature informing key recommendations in this review are from studies utilising the NAFLD nomenclature. For consistency, MAFLD has been used throughout with clarification where required.

## Goals of lifestyle management

The primary objective of lifestyle care is to provide the education, resources and motivation for people with MAFLD to adopt and adhere to lifestyle behaviors that will improve and sustain health and wellbeing. Improving diet quality, increasing physical activity, decreasing or abstinence from alcohol consumption, and smoking cessation can have multifactorial benefits for hepatic and extra-hepatic outcomes [6]. Lifestyle interventions can halt the progression of MAFLD, prevent severe hepatic injury and reverse histological features of MAFLD, and reduce the incidence of hepatocellular carcinoma. They can also improve cardiometabolic disease risk factors to reduce cardiovascular morbidity and mortality and lifestyle-related cancers, which are the leading causes of death in people with MAFLD [6, 7]. From a patient-centred perspective, improving health-related quality of life and patient-important outcomes such as fatigue, energy, mental health, gastrointestinal symptoms and physical function should be key objectives of lifestyle intervention (Table 1). Lifestyle therapy should be integrated into holistic management of MAFLD by the multidisciplinary care team including physicians, dieticians, exercise professionals, psychologists, nursing and other allied health professionals.

**Table 1:**
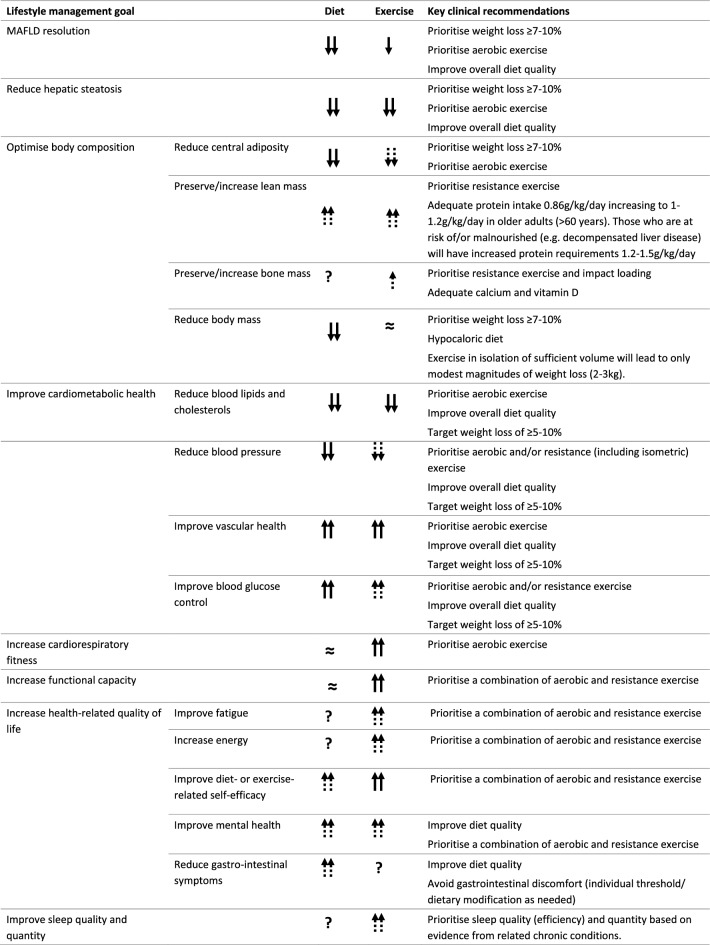
Targets for lifestyle therapy in people with MAFLD

Arrows indicate strength of evidence in MAFLD. Double solid arrow = strong evidence for superior benefit in people with MAFLD; single solid arrow = strong evidence for benefit in people with MAFLD; double dashed arrow = likely superior benefit in people with MAFLD based on evidence from related metabolic conditions; single dashed arrow = likely benefit in people with MAFLD based on evidence from related metabolic conditions; double tilda(≈) = strong evidence for minimal/no benefit; ? = insufficient data

### Weight loss

The metabolic dysfunction that underpins MAFLD is strongly associated with excess and dysfunctional adipose tissue and therefore weight (specifically fat) loss is central to MAFLD management. The magnitude of weight loss is proportional to the improvement in liver-related outcomes [8]. Current guidelines promote weight loss of ≥ 5% for reductions in hepatic steatosis, ≥ 7% for resolution of steatohepatitis and ≥ 10% to regress fibrosis [9, 10]. However, ≥ 7–10% weight loss is often difficult to achieve and sustain with less than one third of MAFLD patients achieving ≥ 5% weight loss with lifestyle intervention over 52 weeks [8], with long-term adherence crucial for sustained benefit. Moreover, around 5–10% of patients with MAFLD are lean with a normal body mass index (BMI, kg/m^2^; < 25 kg/m^2^ in Western populations and < 23 kg/m^2^ in Asian populations), with the highest rates observed among middle-aged people in Asian countries [11]. Current evidence suggests that the severity of liver disease and clinical outcomes in lean patients are akin to those with overweight/obesity [12, 13]. For lean MAFLD, 3–5% weight loss led to resolution of MAFLD in 50% of individuals and can improve cardiometabolic health [14].

### Beyond weight loss

Health and wellbeing benefits have been demonstrated with lifestyle intervention irrespective of weight loss. A ≥ 30% reduction in magnetic-resonance imaging (MRI)-qualified hepatic steatosis was associated with a two-point improvement in the NAFLD Activity Score and resolution of MASH [15], and is considered a clinically meaningful threshold. Whilst there is a strong correlation between weight loss and reduction in hepatic steatosis [16], a ≥ 30% relative reduction in hepatic steatosis is frequently observed with lifestyle interventions with an average weight loss of ≤ 5% [16, 17]. Therefore, whilst weight management should be advised and supported with appropriate behavioural strategies, health targets beyond weight loss (Table 1) should be highlighted with goals individually tailored depending on patient presentation, capabilities and preferences.

## A 24-h integrated lifestyle behavior for MAFLD management

**Fig. 1.**
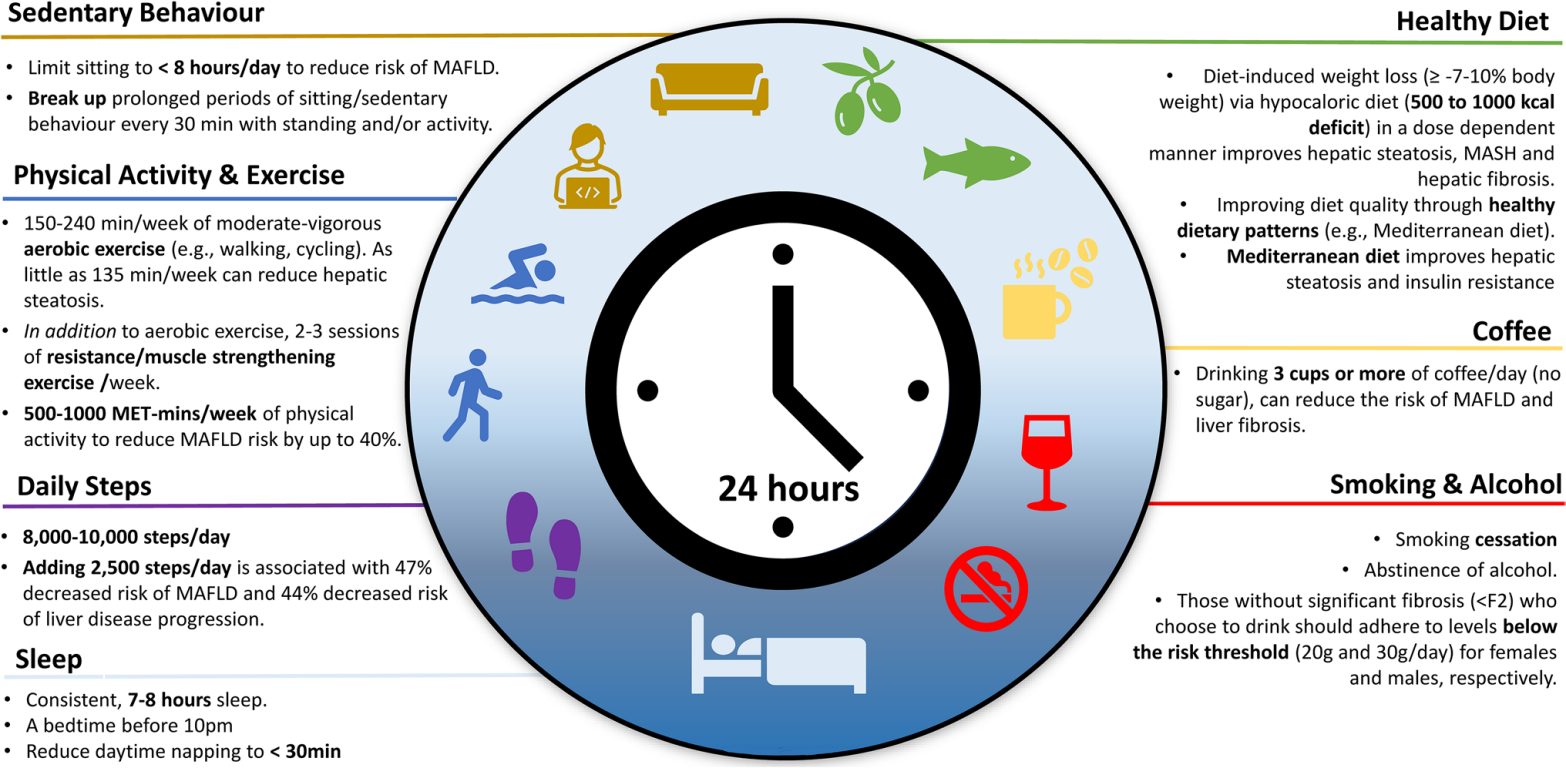
24-h integrated lifestyle behavior recommendations for the management of metabolic dysfunction-associated fatty liver disease. *MAFLD* metabolic dysfunction-associated fatty liver disease, *MET* metabolic equivalent

From a 24-h perspective, the amount of time spent in one type of behavior may influence time spent in another, and therefore lifestyle interventions should consider the entirety of daily movement behaviors (i.e. physical activity, non-exercise activity thermogenesis [NEAT] sedentary behavior and sleep) [18, 19] as well as eating behaviors (i.e. diet quality, quantity and timing) and substance behaviors (i.e. smoking and/or alcohol habits). Whilst the interdependence of integrated lifestyle behavior has not been established in MAFLD [20], lifestyle management should be cognisant of each component and addressed synergistically to improve health (Fig. 1). Depending on the number and nature of lifestyle behaviors to be addressed, a stepped approach to implementation is likely required, with priorities based on shared decision making between the individuals and the multidisciplinary management team.

## Dietary management of MAFLD

**Fig. 2:**
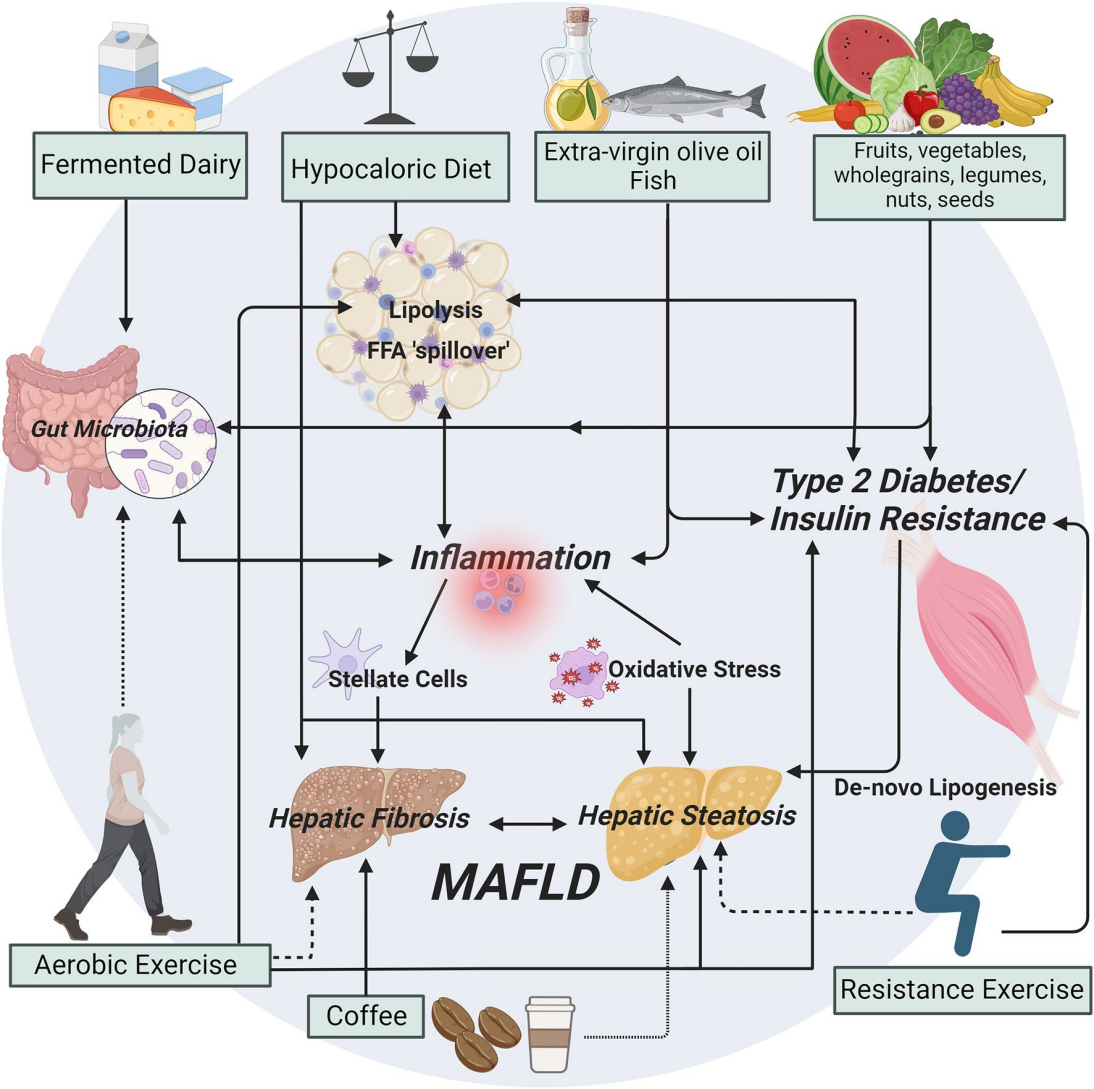
Putative mechanisms for diet and exercise in the management of metabolic dysfunction-associated fatty liver disease. Solid arrows indicate strong evidence of effect; dashed arrows indicate limited or emerging evidence of effect. Created with BioRender.com

Evidence consistently highlights the beneficial effects of improving diet quality (Fig. 1). Dietary recommendations for MAFLD are currently encompassed in clinical management guidelines including those of large international societies [9, 21, 22]. All guidelines caution against alcohol consumption due to its impact on progression to fibrosis and end-stage liver disease, suggesting either adherence to below the risk threshold (20 and 30g/day) for females and males, respectively [21], or recommend abstinence, especially in those with significant fibrosis (> F2) and/or who consume moderate to heavy amounts of alcohol (Fig. 1) [9, 22]. Regarding diet, recommendations prioritise diet-induced weight loss via caloric restriction with acknowledgment of the benefits of improving diet quality which enhance anti-inflammatory and antioxidants to mediate disease onset and progression (Fig. 2). Although recommendations for specific macronutrients composition differ between guidelines, promoting a caloric deficit remains consistent.

### Other dietary considerations

Despite dietary patterns being the central focus of nutrition research there are still a few individual nutrients/foods that are worth highlighting. Management from dieticians which include personalised, tailored dietary advice, based on the current available literature is likely to provide optimal management for people with MAFLD. However, general practical dietary recommendations with associated benefits and contraindications are highlighted in Table 2 which are based on the best available evidence. Evidence suggests that long-term high salt intake independently increases MAFLD risk even after adjusting for total calorie intake. The NHANES data (USA) reported those consuming the highest salt intake were 64% more likely to have MAFLD (defined by hepatic steatosis index) than those reporting the lowest intake (OR 1.46 [1.29, 1.65]) [23]. Furthermore, in light of the metabolic dysregulation of this cohort and the established link between excess salt, hypertension and heart disease it is vital salt intake is monitored and reduced as needed. Evidence also suggests that coffee consumption reduces the risk of hepatic fibrosis and cirrhosis [24, 25]. Coffee consumers are less likely to develop cirrhosis compared to non-consumers (OR of 0.61 [95% CI 0.45–0.84]; 16 studies, 3034 coffee consumers vs 132,000 non-consumers). Coffee consumption can also improve liver enzymes (lower GGT and ALT), reduce hepatic steatosis and fibrosis, and lower the risk of cirrhosis and hepatocellular carcinoma [26]. A dose-dependent response has been observed, indicating coffee consumption > three cups per day reduced MAFLD significantly [27]. Fructose, particularly abundant in processed foods and sugar sweetened beverages is known to exacerbate the risk and progression of MAFLD [28]. In addition to its lipogenic effect, fructose induces inflammation, oxidative and endoplasmic stress contributing to the progression of hepatic steatosis. Finally, there is a growing interest in Ultra-Processed Foods (UPF) which have been associated with increased risk of obesity and T2D [29]. Evidence in MAFLD is also emerging; a recent review demonstrated that both moderate (vs low) (pooled relative risk 1.03 [1.00–1.07]) and high (vs low) (1.42 [1.16–1.75]) UPF intake increased the risk of MAFLD across 60,961 individuals (Table 2) [30].

**Table 2:** Practical lifestyle recommendations based on current, available evidence for MAFLD

	Recommendations	Impacts	Precautions
Diet			
General	Follow healthy dietary patterns (Fig. 1). Most favourable is Mediterranean diet. DASH, TRE, vegetarian/vegan and short-term ketogenic diets may be beneficial	↑MUFA ↓ SFA and free fatty acids ↑Phenolic compounds ↑ dietary fibre, resistant starch, oligosaccharides ↑ ω-3 PUFAs ↑ calcium, phosphorus, magnesium, vitamin D, protein.	Any changes in GI function with dietary changes, should settle after several weeks Eating disorders should be screened and ruled out. Hypoglycaemia management should be provided to those at risk (i) prescribed insulin or sulfonylureas, (ii) those following TRE or Ketogenic diets due to their more restrictive nature. Close monitoring of blood glucose levels due to the rapid and significant dietary changes is recommended. Ketogenic diet should only be followed with recommendation and support from a health care professional and only short term. Conditions that are contraindicated including but not limited to pancreatitis, liver failure, disorders of fat metabolism, primary carnitine deficiency, carnitine palmitoyltransferase deficiency, carnitine translocase deficiency, porphyrias, or pyruvate kinase deficiency. Ketogenic diet may cause upset stomach, dizziness, decreased energy, and mood swings. Intermittent fasting may cause headaches, lethargy, mood swings, and constipation.
Daily/weekly	Use extra virgin olive oil as the main culinary fat Choose wholegrains daily Fruits and vegetables daily Nuts, seeds, legumes and pulses daily Fish, especially oily fish 2–3x/week Poultry and game 2–3x/week Coffee consumers should aim for three or more cups daily Reduce red meat intake to weekly Consume moderate dairy, especially fermented varieties		
Avoid	Ultra-processed foods Processed meat Soft drinks and processed foods high in fructose High-salt foods and adding salt to meals	↑ SFA ↑ Sodium ↑ Fructose ↑ Sucrose	
Exercise			
General	Follow recommendation for PA/exercise and sedentary behavior (Fig. 1)	↑ Cardiorespiratory fitness ↑ Cardiometabolic health ↑ Health-related quality of life ↓ Body adiposity (including visceral adipose tissue). ↓ Hepatic steatosis. ↑ Lean mass.	Review and guidance from a medical practitioner and referral to an appropriately qualified exercise professional is strongly recommended prior to initiating an exercise programme, or significantly changing an existing programme. This enables appropriate screening for contraindications and individual tailoring of the programme and behavioural support strategies. Progressive overload (gradual increases of intensity, duration and/or frequency) of exercise is important to prevent musculoskeletal injury.
Daily/weekly	150–240 min per week of aerobic-based exercise. E.g. 30–60 min of moderate-intensity aerobic exercise (e.g. walking, cycling, jogging, swimming, aerobic dancing) 2–3 days per week of muscle strengthening exercises (e.g. body weight exercises, machine-based resistance exercises, hand weights) Flexibility and/or balance exercises may be incorporated for individuals based on individual goals Look for opportunities to be physically active (e.g. take the stairs, take active breaks)		
Avoid	Prolonged sitting and/or other sedentary behavior (> 30 min at one time; > 8 h a day)		

*DASH* dietary approaches to stop hypertension diet, *TRE* time-restricted eating, *IF* intermittent fasting, *MUFA* monounsaturated fatty acids, *PUFA* polyunsaturated fatty acids, *GI* gastrointestinal, *SFA* saturated fatty acids

### Evidence on dietary patterns

The Mediterranean diet is the most widely researched dietary pattern for MAFLD and promoted as the ‘optimal’ dietary pattern across global clinical guidelines to improve diet quality. There is less research on emerging dietary patterns such as Dietary Approach to Stop Hypertension (DASH) diet, Time Restricted Eating (TRE) and intermittent fasting (IF), ketogenic diet and vegetarian or vegan diets in MAFLD (Table 3). Whilst dietary approaches such as TRE and IF are increasing in popularity, data in MAFLD is limited. All dietary approaches should be undertaken with appropriate oversight of an appropriately qualified health care practitioner (Table 3).

**Table 3:**
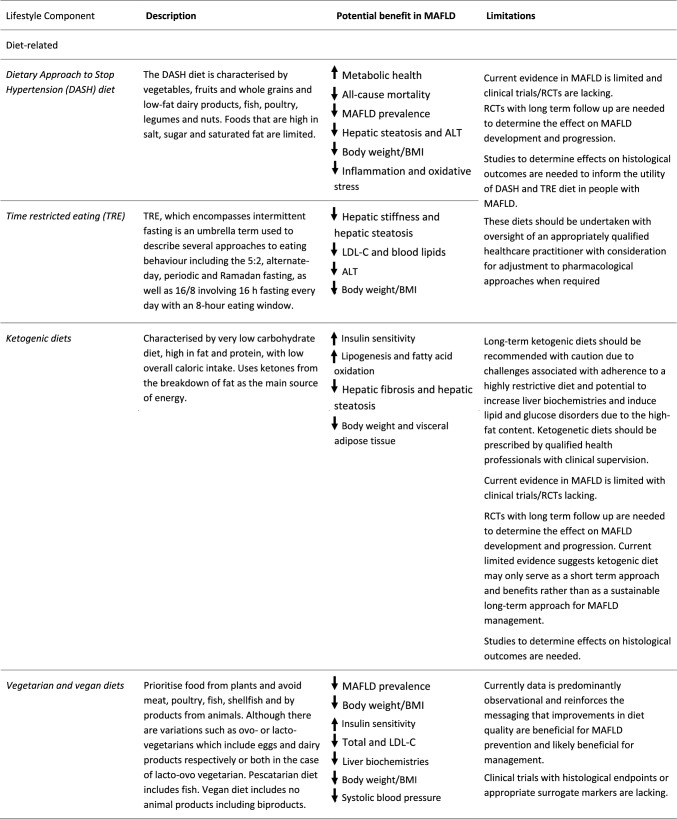

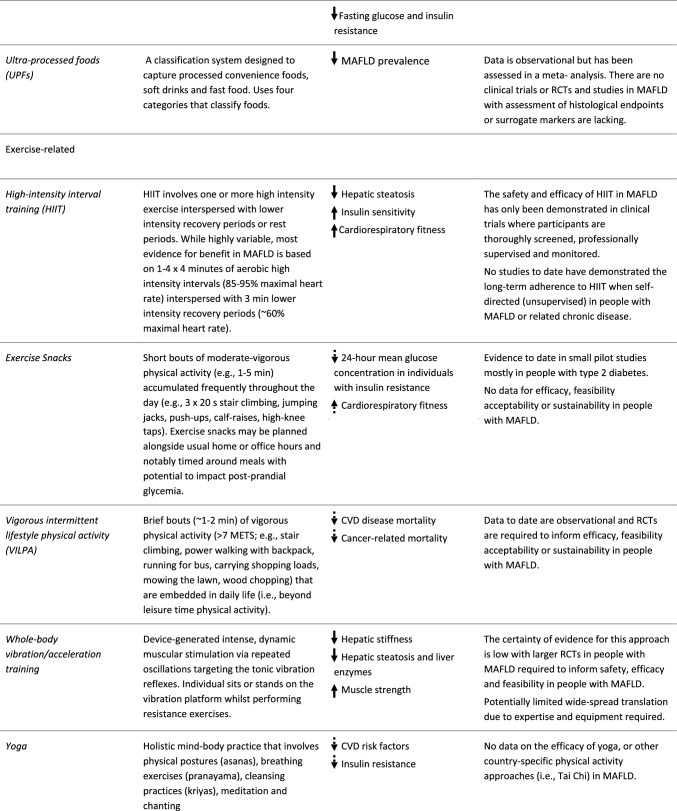
Novel and emerging approaches to lifestyle behavior

Single solid arrow = emerging evidence for benefit in people with MAFLD; Single dashed arrow = likely benefit in people with MAFLD based on evidence from related metabolic conditions

*DASH* dietary approach to stop hypertension, *TRE* time-restricted eating, *MAFLD* metabolic-associated fatty liver disease, *ALT* alanine aminotransferase, *BMI* body mass index, *LCL-C* low-density lipoprotein cholesterol, *RCT* randomised controlled trial, *HIIT* high-intensity interval training, *METS* metabolic equivalents, *CVD* cardiovascular disease

### Mediterranean diet

The research that informs international guidelines highlights the specific benefits of the Mediterranean diet (MedDiet, Box 1) for improving hepatic steatosis and insulin sensitivity. Whilst most of this research has been conducted in Mediterranean regions where the diet is habitual, foundational research has included robust modelling and assessment of the MedDiet for multiethnic populations (e.g. in Australia) and has demonstrated that MedDiet can be adapted to meet sociocultural preferences and is acceptable and effective [31, 32]. Fewer studies have been conducted in non-Mediterranean Western regions, Asia or the Middle East. In Australia, a proof-of-concept crossover trial (*n* = 12, 6 weeks, full meal provision) demonstrated that MedDiet with high compliance led to a mean 39% relative reduction in hepatic steatosis and reduction in insulin resistance [33]. Two subsequent Australian 12-week randomised controlled trials (RCTs) in free living adults demonstrated hepatic and metabolic benefits from both MedDiet and low-fat diet groups and indicated that improving diet quality was beneficial regardless of specific approach [34, 35]. The MedDiet led to a mean 8% absolute reduction in hepatic steatosis, 61% relative reduction in visceral adipose tissue and 0.5 unit improvement in insulin resistance (HOMA-IR) without weight loss in 38 adults with MRI-quantified MAFLD [35]. Whilst weight loss was not imperative for clinically meaningful outcomes in MAFLD, the low-fat diet arm achieved ~ 4% weight loss and greater improvement in hepatic steatosis, visceral fat and insulin resistance (average of − 17%, − 76% and − 1.0 unit), respectively.

In Asian and Middle Eastern populations there have been equivocal findings regarding the adherence to MedDiet and prevalence of MAFLD. In 3,220 Iranian participants (45% with MAFLD), MedDiet adherence was associated with a lower prevalence of MAFLD [36]; however, in 797 Chinese participants (28% with MAFLD) higher MedDiet adherence was inversely associated with hepatic fat content but not MAFLD prevalence [37]. Moreover, there was no association between MedDiet and MAFLD risk in 66,526 participants of the China Multi-Ethnic Cohort Study (16% with MAFLD) [37]. The only RCT in an Asian country compared an Asian modified MedDiet with or without a fatty acid supplement and control, in 88 Chinese females [38]. Reductions in body weight (− 5.3%, − 4.5% and − 2.1%) and MRS-quantified liver fat (− 33%, − 30%, − 10%) were observed in the diet and supplement, supplement only, and control groups, respectively. Collectively, these studies highlight that MedDiet may be beneficial for MAFLD management and diet quality overall is important. However, despite widespread adoption in clinical guidelines, there are relatively few RCTs and there appears to be ethnic differences that should be addressed in clinical guidelines; more consistent benefits of the MedDiet have been shown in Western non-Mediterranean, with equivocal findings in Asia and the Middle East.

Box 1: Definitions for components of integrated lifestyle behaviors

**Table:** 

Lifestyle behavior	Definition
Physical activity	Any bodily movement that elevates the metabolic rate above that of rest and is frequently quantified by the metabolic demand of the activity, or ‘metabolic equivalents or metabolic equivalent of task’ (METs)
Metabolic equivalent of task (METs)	One MET is equivalent to a resting oxygen consumption of 3.5 ml/kg/min and the WHO recommends 500–1000 MET-mins/week. Moderate intensity activity is considered 4.8–7.1 METs and vigorous intensity physical activity is considered 7.2–10.1 METs (for a person with a V̇O_2_max of 40 mL/kg/min)
Sedentary behavior	Behavior involving sitting or lying in a reclined position with minimal energy expenditure (< 1.6 METS)
Exercise	Planned and structured physical activity undertaken to maintain or improve health, wellbeing and/or performance and is generally dichotomised into aerobic- or resistance-based and prescribed at moderate- or vigorous exercise intensities
Non-exercise activity thermogenesis (NEAT)	Energy expenditure of any activity excluding exercise, sleeping or eating (e.g. daily activities, standing, computer work)
Cardiorespiratory fitness	The integrated capacity of the cardiorespiratory system for oxygen transport, uptake and utilisation. expressed as the maximal or peak rate of oxygen consumption (V̇O_2_max or V̇O_2_peak)
Sleep	Recurrent, reversible state vital for health and wellbeing whereby consciousness and mobility are reduced or absent
Mediterranean diet (MedDiet)	Characterised by high intakes of vegetables, fruits, nuts, unrefined cereals, legumes, and olive oil, moderate to high intake of fish, low to moderate intake of dairy products (preferably fermented) and low intakes of saturated fats, red meat and poultry with a regular but moderate consumption of alcohol (mainly wine during meals).

*METs* metabolic equivalent of task, *NEAT* non-exercise activity thermogenesis, *WHO* World Health Organisation, $\dot{V}$*O*_*2*_*max/*$\dot{V}$*O*_*2*_*peak* maximal/peak rate of oxygen consumption

### Novel and emerging dietary patterns

Emerging dietary patterns for the prevention and management of MAFLD include DASH, TRE, ketogenic, vegetarian or vegan diets (defined in Table 3). In broad populations, adherence to DASH diet was associated with improved metabolic outcomes and all-cause mortality [39]. Observational studies have demonstrated lower prevalence of MAFLD with higher DASH adherence [40, 41]. DASH may result in improved hepatic steatosis and cardiometabolic parameters in people with MAFLD who achieve weight loss compared to a general calorie restricted diet [42, 43]. Similarly, RCTs assessing the effect of 5:2 and alternate day calorie restriction (two common approaches to TRE, intermittent fasting is another) have demonstrated improvements in hepatic steatosis, fibrosis, and cardiometabolic health in people with MAFLD [44].A meta-analysis including six studies (4 Ramadan fasting; 2 alternate day fasting, *n* = 417 people with MAFLD), demonstrated a pooled effect favouring intermittent fasting on body weight, BMI and aminotransferases (Table 3) [45].

Ketogenic diets can result in rapid weight loss and reductions in hepatic steatosis in individuals with obesity and MAFLD [46]. Whilst the approach warrants some caution, benefits to liver and cardiometabolic health have been observed in the short-term (Table 3) [47–49]. Observational evidence suggests that vegetarian diets result in 21–57% lower odds of MAFLD and improve cardiometabolic health when compared with non-vegetarians [50]. In people with MAFLD, lacto-ovo-vegetarian or vegan diets led to improvements in body weight, blood pressure and glycemic control [51]. Plant-based healthy diets such as a vegetarian diet were related to a lower MAFLD prevalence and more favorable liver biochemistries [52].

## Physical activity for MAFLD

Physical activity (PA) and sedentary behavior are implicated in the development of MAFLD (Box 1, Fig. 1). Increased PA is associated with a reduced risk of MAFLD in a dose-dependent manner [53–55]. In the UK Biobank, those in the top quartile of PA had a 61% lower risk of developing MAFLD than those the bottom quartile of PA [56]. Moreover, increased daily steps are associated with reduced odds of MAFLD and liver disease progression [56]. Higher volumes of PA may limit the expression of genetic risk factors (PNPLA3, rs738409) for progressive MAFLD [57]. Sedentary behavior and prolonged sitting time are also independent predictors of MAFLD and exhibit dose-dependent relationships [53, 57–59] with an additional hour of sedentary behavior per day correlated with a 4% increased likelihood of MAFLD. Individuals reporting ≥ 7 h of sedentary behavior/day were 34% more likely to have MAFLD than those reporting ≤ 4 h per day [59]. Additionally, sitting for ≥ 8 h/day has been liked to an elevated risk of MAFLD, independent of PA levels [53]. As well as preventing incident MAFLD, moderate–vigorous PA has been associated with MAFLD resolution [60]. In a large cohort of adults, 35% of MAFLD cases at baseline (*n* = 42,536, quantified via ultrasound) resolved at 5-year follow-up. After adjusting for BMI and other potential confounders, moderate–vigorous PA was associated with MAFLD resolution, with the greatest benefit observed with PA frequency ≥ 5 days per week [60]. Concerningly, people with MAFLD spend more time in sedentary behavior and are less likely to meet the recommended physical activity guidelines than those without MAFLD [53, 61], with up to 75–80% of people with MAFLD being insufficiently physically active [62, 63].

### Does PA intensity matter?

The potential intensity-dependent benefits of PA on MAFLD are uncertain and may be contingent on the specific outcome of interest. In 813 people with biopsy-proven MAFLD, only those who self-reported 75min/week of vigorous intensity PA had lower odds for steatohepatitis and only those who self-reported 150 min/week of vigorous intensity PA had lower odds for advanced fibrosis [62]. UK Biobank data (*n* = 840) showed an inverse relationship between PA and hepatic fibro-inflammation (iron corrected cT1), yet for those with elevated hepatic steatosis, only vigorous PA was associated with lower hepatic fibro-inflammation [64]. Moreover, adults with MAFLD who reported at least 50% of their PA as vigorous intensity had a 56% reduction in all-cause mortality and a 79% reduction in cancer-specific mortality than those who were inactive [61]. However, for reduction in hepatic steatosis in people with MAFLD, there does not appear to be an intensity-dependent benefit so long as the recommended volume of PA is met [17].

## Recommendations for exercise prescription

The majority of evidence supporting the benefits of PA for MAFLD are based on trials examining regular, structured exercise (Box 1). Recent guidelines from Australia [17] and the USA [65] have reported strong evidence for the benefits of exercise for people with MAFLD, irrespective of weight loss. Regular exercise may alter the flux of free fatty acids to and from the liver via local and systemic changes to insulin sensitivity and substrate metabolism. These changes may be underpinned by alterations in gene expression (66), gut-liver-axis dysfunction and gut microbiota [67], and the upregulation of peroxisome proliferator-activated receptor gamma and AMP-activated protein kinase [68]. Detailed mechanisms underlying the benefits of exercise on reductions in hepatic steatosis and inflammation are reported elsewhere [17, 57, 69–71]; and summarised in Fig. 2. However, whilst there is strong evidence for benefits on hepatic steatosis, there is a paucity of evidence examining optimal doses of exercise to prevent or reverse liver injury and fibrosis [17]. Pilot work (*n* = 12) demonstrated that 12 weeks of moderate–vigorous intensity aerobic exercise on 3–5 days/week led to a one-stage regression in liver fibrosis in 58% of participants and a one-stage regression in hepatocyte ballooning in 67% of participants [72].

### Aerobic exercise

There is compelling evidence supporting the benefits of aerobic exercise on reducing hepatic steatosis, with absolute reductions of 2–4% (equating to a clinically meaningful ~ 30% relative reduction [15]) observed with exercise of at least moderate intensity for 150–240 min/week [17]. As little as 135 min/week of moderate-intensity aerobic exercise may be effective [17, 73]. Moreover, there does not appear to be additional benefit on hepatic steatosis from increasing the intensity of aerobic exercise. For example, Keating et al. demonstrated reductions in hepatic steatosis with three doses of aerobic exercise that varied in intensity, frequency and duration with no significant differences between those doing (i) 60 min, 4 days/week at 50% $\dot{V}$O_2_peak; (ii) 45 min, 3 days/week at 50% $\dot{V}$O_2_peak or (iii) 45 min, 3 days/week at 70% $\dot{V}$O_2_peak [73]. Similarly, Zhang et al. reported comparable reductions in hepatic steatosis after 6 months of either moderate- or vigorous intensity exercise [26]. Furthermore, comparable reductions in hepatic steatosis are also apparent with high-intensity interval training (HIIT) and moderate-intensity continuous training [74]. Importantly, this level of exercise is likely to concomitantly reduce central adiposity, increase cardiorespiratory fitness and improve cardiometabolic risk factors [17].

### Resistance training

The efficacy of resistance training in isolation on improving MAFLD is less clear. The certainty of evidence is markedly lower compared to aerobic training, primarily due to the heterogeneity in resistance training prescriptions, resulting in disparate outcomes [17]. Whilst some studies have demonstrated significant improvement in hepatic steatosis with resistance training, others have not reported benefit with no clear indications for effective resistance training prescriptions (i.e. intensity, frequency, sets, repetitions etc. [reviewed in [17]). However, resistance training has profound benefits beyond the liver including increasing and maintaining lean mass, improving blood glucose control and blood pressure, and increasing neuromuscular strength and may be particularly beneficial for those with comorbid type 2 diabetes and those with low functional capacity [17]. Given the growing evidence for the interrelationship between sarcopenia and MAFLD pathogenesis,[75] resistance training likely has a vital role in MAFLD management for some individuals. Therefore, resistance training should be considered in addition to achieving the recommended aerobic exercise targets [17], and prioritised in people who are actively losing body mass via diet and/or pharmacological approaches to minimise losses of lean and bone mass and/or in those with sarcopenia.

### Novel and emerging physical activity and exercise approaches:

Emerging PA and exercise approaches for MAFLD are described in Table 3. There is growing evidence for the benefit of HIIT for improving cardiorespiratory fitness and reducing hepatic steatosis in people with MAFLD [74]. However, the suitability and sustainability of HIIT for people with MAFLD beyond controlled laboratory settings is less certain [76]. Novel approaches to achieving the established benefits of more vigorous PA and designed to be embedded within daily activities (thereby reducing the requirement for dedicated leisure time and exercise equipment) include ‘exercise snacks’ and ‘vigorous intermittent lifestyle physical activity’ (VILPA) [77]. Data from the UK Biobank indicated that 3–4 short bouts of VILPA was associated with an up to 40% reduction in all-cause and cancer-related mortality and ~ 50% reduction in in CVD-related mortality. Given these are the main causes of death in MAFLD, VILPA has potential as a feasible and sustainable early PA target for people with MAFLD. Exercise snacks performed 30 min before or after a meal may improve glycemic control in people with insulin resistance or T2D [78, 79]. Meaningful (1 MET) improvements in cardiorespiratory fitness have been observed with 6 weeks of stair climbing efforts (3 × 20 s over 10 min) on three days/week [80]. Finally, whole-body vibration training [81] and other country-specific PA such as Yoga may also be acceptable approaches for aspects of MAFLD management, however, further research is required (Table 3).

### Cardiorespiratory fitness: a ‘vital sign’ for MAFLD

Improved cardiorespiratory fitness is a priority outcome for exercise intervention for MAFLD. Cardiorespiratory fitness (Box 1) is an independent predictor of hepatic steatosis [82], liver fibrosis [83], and histological activity [84, 85]. People with MAFLD have lower cardiorespiratory fitness levels than the general population [83]. In a small study (*n* = 35), 85% of participants with biopsy-proven metabolic-associated steatohepatitis had poor or very poor age- and sex-adjusted cardiorespiratory fitness [84]. Those with most severe disease (NAS > 5). had the lowest $\dot{V}$O_2_peak [84].

Data from the HUNT Study (15,781 adults, 52% female, mean 9.4 years follow up) demonstrated that people with low estimated cardiorespiratory fitness were 17 times more likely to have MAFLD, even if they had low levels of sedentary behavior (≤ 4h/day) [59]. Those with MAFLD and low cardiorespiratory fitness (i.e. 20% least fit per age and sex) had a 52% increased risk of mortality compared to those with high cardiorespiratory fitness (i.e. highest 40%) [59]. There is strong evidence that moderate–vigorous intensity aerobic exercise improves cardiorespiratory fitness in people with MAFLD by an average of 3.6 to 8.3 ml/kg/min across 4 weeks to 8 months [17]. As a 1 MET (3.5 ml/kg/min) improvement in cardiorespiratory fitness has been shown in the general population to reduce all-cause and cardiovascular disease-related mortality by 13% and 15%, respectively [86], this is likely clinically significant.

## Sleep

Few studies have examined the impact of sleep quality and quantity on MAFLD. A review pooled five cross sectional studies and one cohort study demonstrating a 19% increased risk of MAFLD in participants with short sleep duration (< 5–6 h) [87]. This observation was conferred in a larger cohort study with a mean 4-year follow up [88]. People with MAFLD were shown to have poorer sleep efficiency, more frequent daytime napping (> 30 min), higher use of sleeping pills and later wake-up and bedtimes than those without MAFLD [89, 90]. Whilst data suggest that achieving an optimal 7–8 h of sleep and reducing the amount of daytime napping may be beneficial for preventing incident MAFLD (Fig. 1), the impact of sleep on inflammatory processes, hormonally-driven appetite regulation, metabolism and impact of associated fatigue on physical activity and dietary choices warrants further investigation [87].

## Smoking

Smoking is a well-established risk factor for a variety of chronic diseases including CVD and malignancies which are common causes of morbidity and mortality in MAFLD. Smoking is associated with advanced fibrosis in chronic liver diseases like chronic viral hepatitis [91–93]. Evidence has also linked smoking with the risk of MAFLD. A dose-dependent relationship has been suggested between cigarette smoking and the stage of liver fibrosis in patients with MAFLD possibly via its effect on insulin resistance [94]. Indeed, cigarette smoking has also been shown to increase overall mortality in MAFLD in a recent Spanish cohort study [95].

## Barries, enablers and patient perspectives of lifestyle management

Key barriers to the adoption and adherence of healthy lifestyle behaviors in people with MAFLD include perceived lack of time, which may be related to competing priorities such as work and family commitments, cost, access to resources and facilities, lack of social support from friends and family and low self-efficacy for diet and exercise [63, 76, 96–99]. People with MAFLD have indicated a preference for exercise over pharmacotherapies for MAFLD management [98] and perceived holistic benefits including improved fatigue and musculoskeletal symptoms [7, 76]. However, despite excellent adherence to supervised, lab-based, exercise interventions [73, 100, 101], people with MAFLD do not sustain exercise when the supervision is removed [76, 102]. People with MAFLD have reported a lack of provision of information or specific, tailored advice from health care professionals [103]. Combined with a general low awareness of MAFLD, these factors may deprioritise exercise and diet as an integral part of daily life and lower motivation to adopt lifestyle behavior change approaches. Further recommendations for implementation of exercise and diet prescription in clinical practice are detailed elsewhere [17, 65, 104].

## Additional considerations in special populations

### Lean MAFLD

It should be noted that the definition of “lean” is based only on BMI which is a poor surrogate of adiposity and provides no information on visceral obesity. Compared to subcutaneous adipose tissue, visceral adipose tissue (which is directly drained by the portal venous system) is intrinsically more insulin resistant with increased lipolysis and dysregulated adipokine secretion [13, 105]. Indeed, visceral adipose tissue is associated with MAFLD independent of BMI [106, 107]. Further, lean patients have distinct genetic factors and gut microbial profile with increased bile acids and fibroblast growth factor-19 which makes them partially metabolically adapted and helps to keep their total body mass intact [13]. Despite having a normal body weight, people with lean MAFLD have been shown to exhibit lifestyle behaviors that increase cardiometabolic risk, i.e. more sedentary hours and a high-dietary carbohydrate energy ratio with increased intake of fructose and sugary drinks and less PUFAs than lean individuals without MAFLD [108, 109]. Increased carbohydrate energy ratio and less than moderate level of physical activity have been found to be predictors of MAFLD in lean individuals independent of BMI and total energy intake [106]. As such, lifestyle approaches remain the central pillar of management. A healthy, balanced diet with curtailment of free sugars, fructose and soft drinks should be encouraged [110, 111]. Given exercise decreases insulin resistance regardless of weight loss may be particularly relevant for these patients. The rate of remission of MAFLD is proportional to the degree of weight loss in lean patients akin to that in non-lean patients [14]. However, a lower magnitude of weight loss (3–5%) may be sufficient for MAFLD improvement in lean individuals than those with obesity [8].

### MAFLD-cirrhosis

Although patients with MAFLD-cirrhosis usually have overweight/obesity, they frequently exhibit underlying muscle loss (sarcopenia, defined as a significant depletion of muscle mass with functional impairment [112]) with the muscles being partially replaced by fat deposition (myosteatosis). As such, diet in people with MAFLD-cirrhosis and obesity should be individualised emphasising adequate amounts of protein (up to 1.5 g/kg/day) [11, 12]. Sodium restriction is often prescribed in the setting of ascites to around 80 mEq/day. Moreover, whilst there is a paucity of data to inform exercise recommendations for MAFLD-cirrhosis, evidence from broad aetiology compensated cirrhosis suggests that a combination of aerobic and resistance training is feasible and beneficial for improving body composition and functional capacity [113]. In practice, patients with cirrhosis should be encouraged to exercise as per their limits of tolerance under the supervision and guidance of appropriately qualified exercise specialists and the multidisciplinary care team. Given the heightened risks of frailty and low functional capacity, priorities for exercise should focus on increasing/maintaining lean mass, functional status and quality of life [17]. Further recommendations for translation of exercise care into practice for people with cirrhosis are detailed elsewhere [17, 113, 114].

## Access and equality for lifestyle care

A burgeoning challenge for lifestyle care is the insufficient specialised workforce in primary and tertiary care to effectively manage the growing number of people with MAFLD. Coordination of the allied health workforce to collaborate more closely with primary care physicians and specialists is required. For example, expanding the role of pharmacists and other allied health professionals to include identification, referral, patient education and monitoring could meet the growing need for coordinated care [115]. The formation of national policies and programs can streamline care and enable access [116]. Approximately one-third of 102 countries who participated in a global policy review scored zero on the ‘NAFLD policy preparedness index’ [116], and there is a clear need to establish models of care pertinent to local contexts (i.e. culture, environment, services, and systems).

### Digital health

Approaches that embed digital health may increase access to lifestyle care in some regions and may help meet the increased need for coordinated allied health care. Whilst a relatively new area of investigation, interventions have included telephone-based lifestyle counselling, web-based platforms, text messaging and mobile applications, and collectively have demonstrated reductions in body weight, ALT and AST [117]. However, like other approaches to lifestyle intervention, ongoing research and implementation science is required to evaluate strategies to promote long-term adherence to digital health interventions and feasibility of scaled implementation, particularly in regions with poor digital health literacy and/or reduced access to technology.

## Knowledge gaps and future directions

Key knowledge gaps and directions for research enquiry regarding lifestyle management for MAFLD are outlined in Table 4. In the context of a 24-h integrated approach to lifestyle management of MAFLD, strong evidence exists for diet and exercise approaches, smoking cessation, and reducing or abstaining from alcohol. There are less data to inform approaches for sleep and their integrated relationship in MAFLD. Whilst there is emerging evidence for time-restricted eating, the impact of diet and exercise timing is largely unknown. Large, multidisciplinary randomised controlled trials with adequate follow-up and robust documentation of diet and exercise adherence, specifically in those diagnosed with MAFLD under the new criteria, who are more metabolically complex, are required. Trials implementing and assessing the efficacy of lifestyle interventions that emphasise sustained behavior change in MAFLD to inform strategies for adherence and long-term weight loss maintenance are also needed. Interventions should be co-designed with people with MAFLD and their care stakeholders to embed their perspectives. Finally, genetic and epigenetic factors that predispose to MAFLD development have been identified and may explain in-part the heterogeneity in response to lifestyle intervention. For example, dietary approaches have shown to be more effective in reducing hepatic steatosis in those carrying the PNPLA 3 I148M single nucleotide polymorphism [118]. Pilot data (*n* = 18) indicated a greater reduction in hepatic steatosis in those with rs738409 PNPLA3 homozygosity (45%) than those with the CC following a 6 day hypocaloric, low-carbohydrate diet [119]. Precision medicine approaches for lifestyle therapy are likely to emerge with ongoing research and technological advances [120].

**Table 4:** Key knowledge gaps and future directions

Future research direction	Research gaps in people with MAFLD
Diet	Safety, feasibility and efficacy of emerging dietary patterns such as time-restricted eating and consideration surrounding timing of meals and snacks which has not been assessed to date Standardised methods and tools for data collection and reporting for diet prescription and adherence Feasibility and efficacy of Mediterranean eating approaches in Western and non-Mediterranean populations Effect of dietary interventions on liver fibrosis and metabolic-associated steatohepatitis resolution, including long-term sustainability of benefits
Exercise	Safety, feasibility and efficacy of exercise in people with compensated and decompensated MAFLD-cirrhosis Relative benefits of nuanced exercise timing (e.g. timing of exercise relative to meals or chronotype) Standardised methods and tools for data collection and reporting for physical activity/exercise and exercise adherence Effect of exercise on liver fibrosis and metabolic-associated steatohepatitis resolution including long-term sustainability of benefits Efficacy of exercise (specifically, resistance training) to maintain fat-free mass in people with MAFLD achieving massive weight loss through diet, surgery or pharmacotherapeutics (e.g. GLP-1 agonists)
Sleep	Interventions to improve sleep quantity and quality and whether changes in sleep behavior can improve and regress MAFLD
24-hour integrated lifestyle behavior	Integrated relationship between physical activity, sedentary behaviors, NEAT, sleep and diet: to create an individually tailored hierarchy of behavior Tailored, integrated multi-modal lifestyle interventions Integrated allied health model of care ‘Precision medicine’ approaches to lifestyle intervention
Lifestyle care reach and sustainability	Safety, efficacy/effectiveness and feasibility of digital health and technology to deliver, monitor, manage and motivate for lifestyle care Low cost and accessible approaches to lifestyle care

*MAFLD* metabolic dysfunction-associated fatty liver disease, *NEAT* non-exercise activity thermogenesis

## Conclusions

A healthy lifestyle is the forefront of management for MAFLD and co-existing metabolic conditions. Current clinical management guidelines emphasise the importance of weight loss for individuals with MAFLD with overweight or obesity. Mediterranean dietary pattern may be optimal for MAFLD although studies in non-Mediterranean regions are limited. However, with adaptation to sociocultural preferences and local food systems, this approach is likely beneficial. Whilst it is prudent to recommend reductions in sedentary behavior and disrupting sitting time to complement increased physical activity, supporting individuals to attain the recommended regular structured exercise should be prioritised. Future guidelines should consider the heterogeneity of the MAFLD diagnosis within clinical management guidelines requiring careful consideration of the increased and growing MAFLD population and the public health implications posed.

## Data Availability

Not Applicable.
